# Fast and robust adjustment of cell mixtures in epigenome-wide association studies with SmartSVA

**DOI:** 10.1186/s12864-017-3808-1

**Published:** 2017-05-26

**Authors:** Jun Chen, Ehsan Behnam, Jinyan Huang, Miriam F. Moffatt, Daniel J. Schaid, Liming Liang, Xihong Lin

**Affiliations:** 1 Division of Biomedical Statistics and Informatics, Department of Health Sciences Research and Center for Individualized Medicine, Mayo Clinic, 200 1st St SW, Rochester, MN 55905 USA; 2 State Key Laboratory of Medical Genomics, Rui-jin Hospital & Shanghai Jiao Tong University School of Medicine, 197 Rui Jin Er Road, Shanghai, 200025 China; 3 Faculty of Medicine, National Heart & Lung Institute, Imperial College London, Dovehouse St, London, SW3 6LY UK; 4 Department of Epidemiology, Harvard T.H. School of Public Health, Boston, 677 Huntington Ave, Boston, MA 02115 USA; 5 Department of Biostatistics, Harvard T.H. School of Public Health, 677 Huntington Ave, Boston, MA 02115 USA

**Keywords:** Epigenome-wide association, cell mixture, surrogate variable analysis, DNA methylation

## Abstract

**Background:**

One problem that plagues epigenome-wide association studies is the potential confounding due to cell mixtures when purified target cells are not available. Reference-free adjustment of cell mixtures has become increasingly popular due to its flexibility and simplicity. However, existing methods are still not optimal: increased false positive rates and reduced statistical power have been observed in many scenarios.

**Methods:**

We develop SmartSVA, an optimized surrogate variable analysis (SVA) method, for fast and robust reference-free adjustment of cell mixtures. SmartSVA corrects the limitation of traditional SVA under highly confounded scenarios by imposing an explicit convergence criterion and improves the computational efficiency for large datasets.

**Results:**

Compared to traditional SVA, SmartSVA achieves an order-of-magnitude speedup and better false positive control. It protects the signals when capturing the cell mixtures, resulting in significant power increase while controlling for false positives. Through extensive simulations and real data applications, we demonstrate a better performance of SmartSVA than the existing methods.

**Conclusions:**

SmartSVA is a fast and robust method for reference-free adjustment of cell mixtures for epigenome-wide association studies. As a general method, SmartSVA can be applied to other genomic studies to capture unknown sources of variability.

**Electronic supplementary material:**

The online version of this article (doi:10.1186/s12864-017-3808-1) contains supplementary material, which is available to authorized users.

## Background

The development of array-based DNA methylation profiling technologies, such as Illumina Infinium HumanMethylation450 BeadChip, has enabled large-scale epigenome-wide association studies (EWAS). Such studies seek to identify CpG methylation variants that are associated with diseases or exposures [1, 2]. Unlike DNA sequences, DNA methylation is cell type-specific. Consequently, cell type heterogeneity plays a confounding role in identifying differentially methylated CpG positions (DMPs). Results might be driven by differential cell mixtures rather than cell type-specific relationships with disease or exposure [3–6]. Therefore, proper adjustment of differential cell populations in EWAS is critical in reducing false associations. Several statistical methods have been proposed to adjust for cell mixtures. They can be classified into reference-based and reference-free methods [7]. Reference-based methods require a reference panel of purified cell types to identify cell-type-specific DMPs, which are then used to infer cell proportions [8]. However, if the reference panel consists of cell types different from the study samples, or the methylation data are subject to large measurement errors, the accuracy of the inferred cell proportions will be affected accordingly. Moreover, a reference panel may not be available for some tissue types such as cancer tissues, which limits the use of this approach. To address the above limitations, reference-free methods have been proposed, including RefFreeEWAS [9], FaST-LMM-EWASher [10], ReFACTor [11] and others [12–15]. A recent evaluation study found that RefFreeEWAS and FaST-LMM-EWASher are subject to high false positive rates or poor statistical power [7]. ReFACTor, a recent method that relies on principal component analysis (PCA) of a subset of informative CpG sites, has been shown to be significantly more powerful than previous reference-free methods especially when the signal is sparse. However, as common to PCA-type methods, it has the potential problem of overcorrection, and hence loss of power, when there are many phenotype-associated DMPs [11]. A large number of DMPs have been observed in many studies such as EWAS on age and cancer [16–18]. Thus protecting the statistical power in such dense-signal scenarios should be an essential part of cell mixture adjustment for EWAS.

We show that none of the current popular reference-free methods is robust across biologically relevant scenarios. To address the limitation of current methods, we present SmartSVA, an optimized version of surrogate variable analysis (SVA) method [19, 20], for reference-free adjustment of cell mixtures. Though the traditional SVA has been shown to be relatively robust by a recent evaluation study [7], we show that it fails to control for false positives when there is strong confounding due to cell mixtures. The drawback of SVA is due to its failure to reach convergence using a fixed number of iterations. SmartSVA improves its ability to control for false positives by explicitly imposing a convergence criterion. Furthermore, SmartSVA is an order-of-magnitude faster than traditional SVA due to algorithmic improvements.

## Results

### Simulation strategy and performance evaluation

**Fig. 1:**
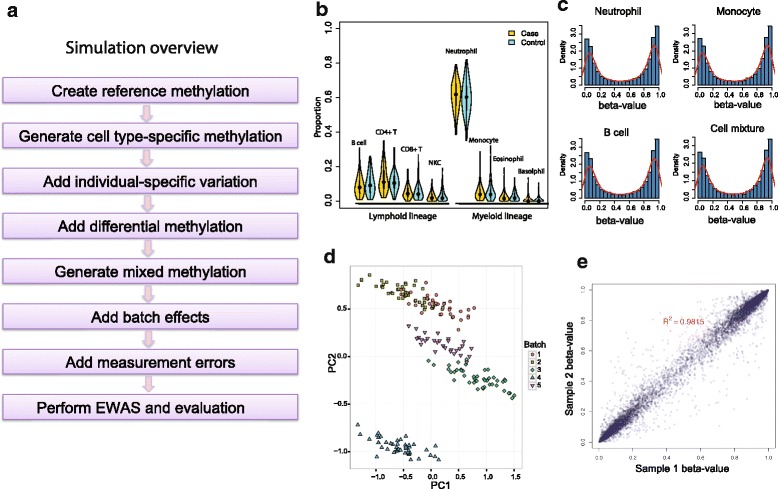
An overview of the simulation strategy. The distributions of cell proportions and methylation values are simulated to reflect the real data with various sources of variability. **a** The flow chart of the simulation process. **b** The distribution of the leukocyte subtype proportions generated using a Dirichlet distribution. **c** The bimodal distribution of methylation beta-values generated using a three-component mixture model. **d** Simulated batch effects cluster the samples into distinct groups. Principal component analysis (PCA) on the methylation beta-values was used to project the samples onto the first two principal components (PCs). **e** Correlation between two biological replicates reflecting the variability due to various sources

We evaluated the performance of SmartSVA by comparing to other reference-free methods using realistic simulations (Fig. 1). Figure 1a gives an overview of the simulation process, where we added various sources of variability (see “Methods” for more details). To reflect the full complexity observed in real tissue samples, we simulated eight cell types from two lineages mimicking the blood leukocyte mixtures (Fig. 1b). A bimodal distribution of methylation data was realized by using a three-component mixture model (Fig. 1c). Batch effects were added to the samples from the same batch (e.g. bisulfite conversion plate) to create the clustering pattern of the samples usually observed in real data (Fig. 1d). We also added individual-specific methylation variation and measurement errors to recapitulate the methylation profiles observed in real data (Fig. 1e). The key parameter values were based on the estimates from real methylation data of purified leukocytes (Additional file 1: Note 1 and Table S1). To conduct a comprehensive evaluation, we simulated scenarios with different levels of confounding due to cell mixtures and different numbers of DMPs. The ability to control for false positives (type I error) was assessed using the genomic inflation factor (λ) on the non-DMPs as well as the observed false discovery rate (FDR) and family-wise error rate (FWER) when FDR control (Benjamini-Hochberg procedure) and Bonferroni correction were applied at 5% level. Power was assessed by the true positive rate after multiple testing correction. The assessment reflects statistical procedures usually employed in EWAS.

### Simulation results

**Fig. 2:**
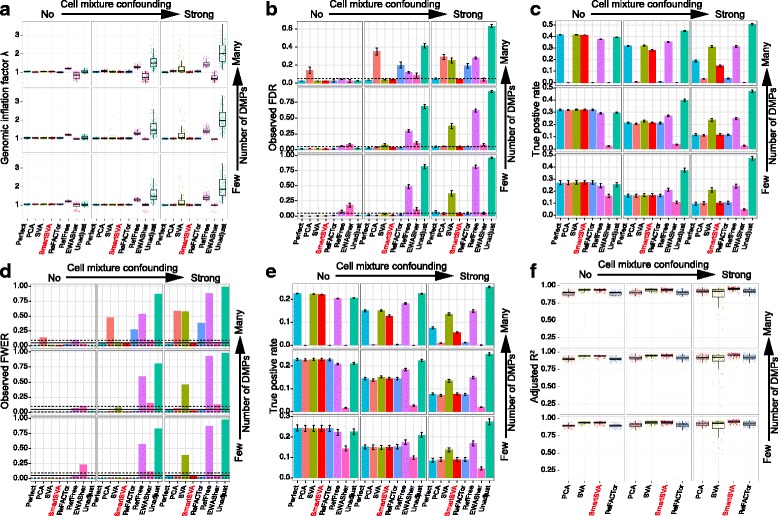
Performance comparison of reference-free cell mixture adjustment methods based on simulated data and binary phenotypes. The samples were randomized into five batches, where random methylation differences were added to each batch. Nine scenarios were investigated with different levels of signal density (0.1%, 1% and 10%) and cell mixture confounding (no, moderate and strong). The “Perfect” method, which adjusts for real cell proportions and batch effects, is included for benchmarking purposes. **a-f** Performance was evaluated by (**a**) Genomic inflation factor λ on non-DMPs, (**b-c**) the observed false discovery rate (FDR) and true positive rate after FDR control (5% level, dashed line), (**d-e**) the observed family-wise error rate (FWER) and true positive rate after Bonferroni correction (5% level, solid line; 95% CI, dashed lines) and (**f**) the fraction of cell compositional variability (8 cell types jointly) explained by the components (PC/SV) as quantified by adjusted R^2^. Error bars represent the standard errors. The SmartSVA is the only method that controls the type I error under the nominal level across scenarios and retains power in dense signal scenarios

**Fig. 3:**
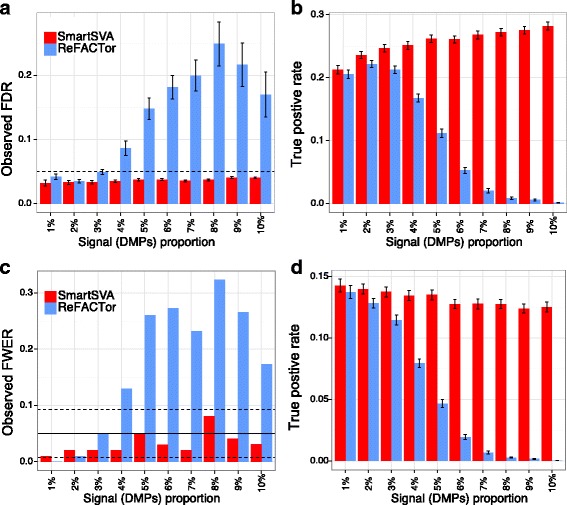
The power of ReFACTor decreases with increasing signal densities. Moderate cell mixture confounding was simulated in this example. Performance was evaluated by (**a-b**) the observed false discovery rate (FDR) and true positive rate after FDR control (5% level, dashed line) and (**c-d**) the observed family-wise error rate (FWER) and true positive rate after Bonferroni correction (5% level, solid line; 95% CI, dashed lines). The number of components for ReFACTor was estimated based on RMT. As we increase the signal proportion, the power of ReFACTor decreases significantly, together with reduced ability to control for false positives. In contrast, SmartSVA is very robust and retains the power irrespective of the signal proportions

**Fig. 4:**
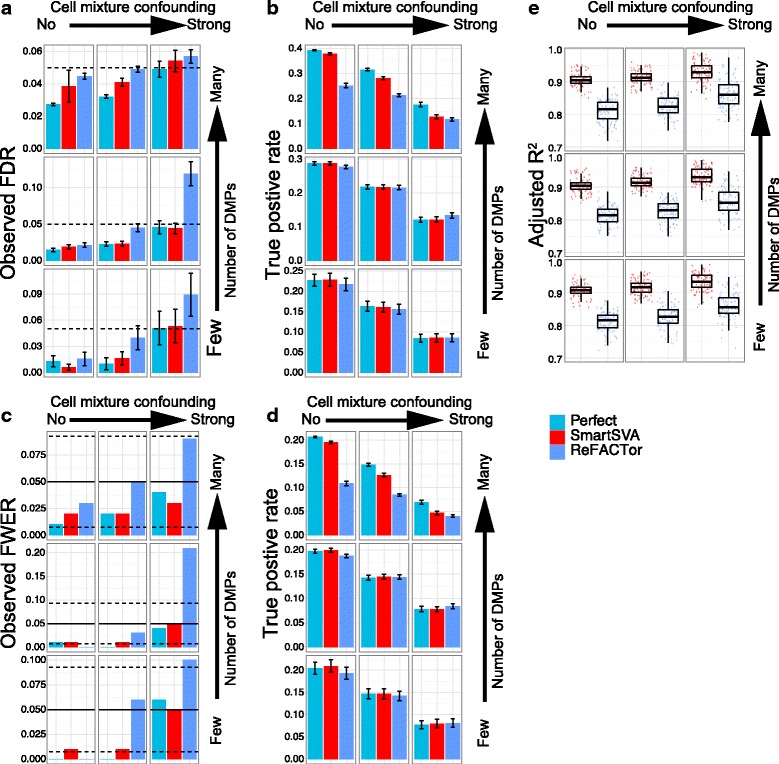
The performance of ReFACTor with site selection on the control samples. Nine scenarios were investigated with different levels of signal density (0.1%, 1% and 10%) and cell mixture confounding (no, moderate and strong). The “Perfect” method, which adjusts for real cell proportions and batch effects, is included to benchmark other methods. Performance was evaluated by (**a-b**) the observed false discovery rate (FDR) and true positive rate after FDR control (5% level, dashed line), (**c-d**) the observed family-wise error rate (FWER) and true positive rate after Bonferroni correction (5% level, solid line; 95% CI, dashed lines) and (**e**) the fraction of cell compositional variability (8 cell types jointly) explained by the components as quantified by adjusted R^2^. Error bars represent the standard errors. The SmartSVA is still more powerful than ReFACTor in dense signal scenarios and captures the cell composition better than ReFACTor. The type I error for ReFACTor is inflated in highly confounded scenarios, indicating less efficient site selection using half of the samples

We first conducted case–control based simulations, where we varied the cell compositions and the number of DMPs between cases and controls to create different levels of confounding effects and signal densities. We averaged the results over 100 simulation runs and assessed the performance of eight competing methods based on (1) genomic inflation factor λ (Fig.2a), (2) the observed FDR and true positive rate after FDR control (Fig. 2b,c), (3) the observed FWER and true positive rate after Bonferroni correction (Fig. 2d,e), and (4) the percentage of cell composition variance explained (*R* ^2^, Fig. 2f). Clearly, as the cell mixture confounding became stronger, the statistical power was reduced for all the methods and type I error inflation became more prominent for some methods. As expected, the unadjusted method (green color), which did not correct for cell mixtures, had the worst type I error control in the presence of cell mixture confounding. Consistent with a previous report [7], RefFreeEWAS tends to have the highest power, but it did not control for false positives very well for confounded scenarios as indicated by an inflated λ, observed FDR and FWER. On the contrary, FaST-LMM-EWASher, which aims to control theλover all CpGs, was very conservative and had the lowest power especially when the signal was dense. As the number of DMPs increases, an inflated λover all CpGs is expected [21] (Additional file 1: Figure S1 and Table S2) and forcing the overallλto 1 could potentially lead to loss of power and deflation of λover non-DMPs (Fig. 2a). Interestingly, the classic PCA method performed quite well when the DMP signal was not very dense. As the signal became denser, PCA became unstable and powerless.

Overall, the recently proposed ReFACTor had better performance than previous reference-free methods. However, the performance of ReFACTor was sensitive to the number of principal components used. The default setting for ReFACTor (k = 5) was not sufficient to control for false positives in confounded scenarios (Additional file 1: Figure S2). We thus increased the number of components to that based on random matrix theory [13], which generally controlled for false positives except for some scenarios (Fig. 2b,d). ReFACTor worked well when the signal was not very dense, but it suffered substantial power loss in the presence of many DMPs since the top components could capture these DMPs (Fig. 2c,e). The power loss was also coupled with slightly increased type I error rate. Thus, in such scenarios, ReFACTor recovered few DMPs with less accuracy. The decrease of power for ReFACTor became apparent when there were more than 1% DMPs (Fig. 3). To mitigate the problem in case–control studies, we next performed the site selection on the control samples. With this strategy, the power was improved significantly but was still lower than SmartSVA, probably due to a less efficient capture of cell mixtures using only half of the samples for site selection (Fig. 4). It is also less clear how to extend the strategy to continuous phenotypes such as age. Taking residuals by regressing out the phenotype effect or removing components mostly associated with the phenotype will not solve the problem for PCA-based methods including ReFACTor (Additional file 1: Note2).

In contrast, SmartSVA was robust across scenarios: it controlled for the type I error rates, and its power was comparable to the “Perfect” method, where the real cell proportions were adjusted (Fig. 2). Since the “Perfect” method represents the optimal adjustment procedure, the performance of SmartSVA was nearly optimal in these simulated scenarios. Besides the 5% level usually used for FDR control, we further evaluated the type I error control of SmartSVA at various FDR levels (1%–20%). We observed that SmartSVA could control the FDR under the nominal level for all scenarios (Additional file 1: Figure S3). As a comparison, the type I error control of the traditional SVA was poor in highly confounded scenarios (Fig. 2, Additional file 1: Figure S3). The bad performance was due to the failure to reach convergence using a fixed number of iterations. Through an explicit convergence criterion, the surrogate variables constructed by SmartSVA could better capture the cell composition than traditional SVA as demonstrated by a higher percentage of cell composition variation explained (Fig. 2f). Application to a real data set with known blood cell counts showed a comparable performance of SmartSVA and ReFACTor in explaining the cell proportion variability (*n* = 357, Additional file 1: Figure S4) [22]. SmartSVA also retained statistical power when the signal was dense, a property not enjoyed by ReFACTor and PCA.

We also compared to RefFreeCellMix [15], which was the most recent reference-free method based on non-negative matrix factorization. Though RefFreeCellMix had a higher power than PCA and ReFACTor using the same number of components, significant type I error inflation was observed in confounded scenarios (Additional file 1: Figure S2). To bring down the type I error rate close to the nominal level, more components were required. However, increasing the number of components was concomitant with the decrease in power. As a PCA-type method, RefFreeCellMix has the same problem of over-correction as PCA and ReFACTor in dense signal scenarios due to the capture of the signal by some top components.

**Fig. 5:**
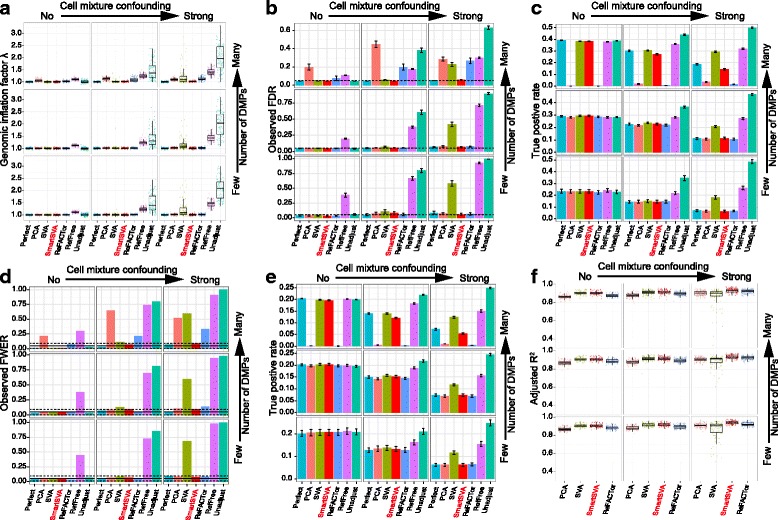
Performance comparison of reference-free cell mixture adjustment methods based on simulated data and continuous phenotypes. The samples were randomized into five batches, where random methylation differences were added to each batch. Nine scenarios were investigated with different levels of signal density (0.1%, 1% and 10%) and cell mixture confounding (no, moderate and strong). The “Perfect” method, which adjusts for real cell proportions and batch effects, is included for benchmarking purposes. **a-f** Performance was evaluated by (**a**) Genomic inflation factor λ on non-DMPs, (**b-c**) the observed false discovery rate (FDR) and true positive rate after FDR control (5% level, dashed line), (**d-e**) the observed family-wise error rate (FWER) and true positive rate after Bonferroni correction (5% level, solid line; 95% CI, dashed lines) and (**f**) the fraction of cell compositional variability (8 cell types jointly) explained by the components (PC/SV) as quantified by adjusted R^2^. Error bars represent the standard errors. The SmartSVA is the only method that controls the type I error under the nominal level across scenarios and retains power in dense signal scenarios

To rule out the possibility that the superior performance of SmartSVA was due to capturing the simulated batch effects in addition to the cell mixtures, we next performed additional experiments without simulating batch effects. The results remained very similar (Additional file 1: Figure S5). We finally simulated continuous phenotypes. SmartSVA was still very robust and performed better than the competing methods (Fig. 5).

### Runtime comparison

**Fig. 6:**
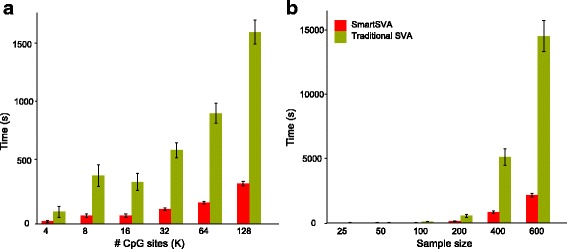
Runtime comparison of SmartSVA and traditional SVA based on a real data set. We subsampled the data set to study the scalability of SmartSVA with CpG number (**a**) and the sample size (**b**)

SmartSVA is also computationally more efficient than the traditional SVA due to algorithmic improvement. We compared the runtime of SmartSVA to traditional SVA across different numbers of CpGs and sample sizes by subsampling a real data set [1]. SmartSVA improved the computation speed by almost a factor of 10 and the computational advantage was more pronounced with increasing sample sizes (Fig. 6), making SmartSVA suitable for large-scale EWAS.

### Real data applications

Finally, we evaluated the performance of SmartSVA on two real data sets from association studies of gastric cancer and human aging [23, 24]. These two data sets hypothetically represent the most complicated scenario of dense signal and strong confounding. Since currently there are no real gold standard datasets with all the DMPs known, we focused on studying (1) whether the results were consistent with those from the simulation studies, and (2) whether the identified DMPs were biologically interpretable.

We compared SmartSVA to various reference-free methods and the results were shown in Table 1. Unadjusted analyses had serious genomic inflation (λ = 25.8 and 7.73), and the estimated proportions of non-DMP were very small (*π*_0_ =0.18 and 0.35), indicating potential strong confounding in these data sets. Random matrix theory estimated 20 and 68 components for these two data sets (see “Methods”), suggesting that there might be many unknown sources of variation besides the cell mixtures affect the methylation. We thus used these numbers of components in the regression model to adjust for cell mixtures and other batch effects for both traditional SVA and SmartSVA. Surprisingly, application of traditional SVA to the gastric cancer data set resulted in an even larger genomic inflation factor than the unadjusted procedure. Such a high inflation factor is probably due to the capture of sources of variability other than cell mixtures, which increases the statistical power to detect cell mixture-related confounding signals. Application to the age data set reduced the genomic inflation but the inflation factor was still very large, compared to that from SmartSVA (λ = 2.98 vs 1.33). The behaviors of the traditional SVA on these two data sets were consistent with the observations from the simulation studies, and thus its use in highly confounded scenarios was not recommended. The results of RefFreeEWAS and FaST-LMM-EWASher were also consistent with the simulations: RefFreeEWAS recovered far more DMPs than SmartSVA while FaST-LMM-EWASher was the least powerful and recovered the fewest DMPs. The increased power of RefFreeEWAS should be interpreted cautiously since the type I error was substantially elevated. For ReFACTor, we again found that the results were sensitive to the number of components (Additional file 1: Figure S6). We thus used the number of components estimated from RMT for a fair comparison. ReFACTor was very conservative: it recovered only 1 and 177 Bonferroni-significant DMPs for the two data sets respectively, compared to 30 and 679 DMPs for SmartSVA. The reduced power of ReFACTor was consistent with its performance in dense signal scenarios.

**Table 1:** EWAS summary statistics for two real data sets. FDR control and Bonferroni correction were used for selecting DMPs

Data set	Method	*π*_0_^a^	*λ* ^*b*^	#(q < 0.05)^c^	#(p_b_ < 0.05)^d^
GSE30601 (Gastric cancer, 27 K)	Unadjusted	0.18	25.8	21,487	8,323
	SVA^e^	0.13	59.3	23,404	13,846
	SmartSVA	0.75	1.72	888	30
	RefFreeEWAS	0.70	2.03	1,266	68
	EWASher^f^	1.00	0.87	3	1
	ReFACTor	0.95	1.07	23	1
GSE40279 (Human aging, 450 K)	Unadjusted	0.35	7.73	245,279	41,357
	SVA	0.60	2.98	102,509	20,644
	SmartSVA	0.87	1.33	5,620	679
	RefFreeEWAS	0.68	2.20	43,791	5,192
	ReFACTor	0.91	1.23	1,577	177

^a^ *π*_0_ is the percentage of non-DMP estimated based on “qvalue” method

^b^ *λ* is the genomic inflation factor calculated on all CpGs

^c^ FDR control is based on “qvalue” method and 5% level

^d^Bonferroni correction was used at 5% level

^e^The classic SVA with default implementation was used (B = 5)

^f^FaST-LMM-EWASher was performed without filtering out consistently methylated or unmethylated CpGs. For the age data set, we were unable to obtain the results within one week

**Fig. 7:**
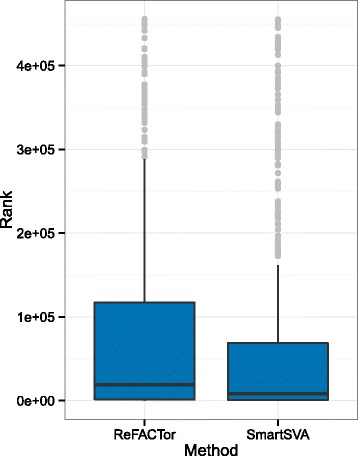
Comparison of the distribution of the ranks of the 583 age-associated CpGs for ReFACTor and SmartSVA. These age-associated DMPs were selected with support from two independent age-association studies based on purified CD4+ T-cells. SmartSVA achieves a lower rank than ReFACTor

We next look more closely at the recovered DMPs. To evaluate the age-associated DMPs recovered by SmartSVA, we curated a list of highly confident age-associated DMPs with support from two independent age association studies using purified CD4+ T-cells [16, 17]. We included CpG probes that passed Bonferroni correction in both studies, resulting in a total of 583 age-associated DMPs (CD4+ aDMPs). Among the 679 Bonferroni-significant DMPs recovered by SmartSVA, 130 probes were on the list. Clearly, these DMPs were enriched in CD4+ aDMPs (136-fold enrichment, p < 2.2e-16). Interestingly, the 57 out of the177 DMPs recovered by ReFACTor were also in the list (248-fold enrichment, *p* < 2.2e-16). We then compared the ranks of the P values of these CD4+ aDMPs for the two methods (Fig. 7). SmartSVA achieved a much lower median rank of 8,028 (top 1.8%), compared to 18,834 for ReFACTor (top 4.1%). Taken together, both SmartSVA and ReFACTor seemed to recover real signals, but SmatSVA was more powerful than ReFACTor to identify CD4+ aDMPs. For the gastric cancer-associated DMPs recovered by SmartSVA (q < 0.05), gene set enrichment analysis did reveal an enrichment of cancer-related pathways (Table 2). Therefore, the DMPs recovered by SmartSVA were biologically interpretable and the increased statistical power was probably not a result of false positives.

**Table 2:** Top 10 enriched KEGG pathways and GO biological processes^a^

	Count	%	P-Value^b^
KEGG Pathway			
Chemokine signaling pathway	13	2.5	0.021
Endometrial cancer	6	1.2	0.028
Melanoma	7	1.4	0.03
Bladder cancer	5	1	0.049
Cytokine-cytokine receptor interaction	15	2.9	0.052
Thyroid cancer	4	0.8	0.07
Jak-STAT signaling pathway	10	1.9	0.072
Prostate cancer	7	1.4	0.075
Pancreatic cancer	6	1.2	0.09
Chronic myeloid leukemia	6	1.2	0.1
GO Biological Process (level 5)			
regulation of apoptosis	39	7.6	0.003
regulation of programmed cell death	39	7.6	0.0036
catechol catabolic process	3	0.6	0.0083
telomere maintenance	5	1	0.0089
positive regulation of T cell mediated cytotoxicity	3	0.6	0.017
positive regulation of apoptosis	22	4.3	0.018
positive regulation of programmed cell death	22	4.3	0.019
positive regulation of cell death	22	4.3	0.02
positive regulation of hormone secretion	5	1	0.021
nucleic acid transport	8	1.6	0.025

^a^The 29 Bonferroni-significant, gastric cancer-associated DMPs recovered by SmartSVA were used as an input to DAVID Functional Annotation Bioinformatics Microarray Analysis (https://david.ncifcrf.gov/)

^b^A modified Fisher Exact P-Value based on EASE Score for gene-enrichment analysis (https://david.ncifcrf.gov/). Usually P-Value equal or smaller than 0.05 is to be considered strongly enriched in the annotation categories

## Discussion

Addressing confounding due to cell mixtures in EWAS is critical for moving the field forward [4–6]. There are a plethora of methods for cell mixture adjustment for EWAS, and new methods continue to be published [7]. An ideal method should be robust in the sense that it should control type I errors under the nominal level across various scenarios. Otherwise, the statistical significance of the recovered associations would be difficult to assess and the reported P values would not reflect the true significance. It should also be statistically powerful so that its power is not severely compromised by controlling the type I error. The consequences of the application of a non-robust algorithm are either increased type I error rate or reduced statistical power, casting doubt on the identified associations. To evaluate the robustness of a proposed method, comprehensively simulation studies covering as many scenarios as possible are needed before applying it to real data sets.

We demonstrated that SmartSVA was more robust than the competing methods across a wide range of biologically relevant scenarios. Among the methods evaluated, SmartSVA is the only method that could control the type I error under the nominal level and retain the power close to the “Perfect” procedure. Most remarkably, when there is a dense signal, SmartSVA could still have power while ReFACTor and classic PCA become powerless due to overcorrection. Since widespread DNA methylation change is not a rare phenomenon for EWAS [16–18], it is safer to use methods that do not rely on sparse signal assumption. As a reference-free method, SmartSVA can be applied to any tissue types such as cancer tissues without the need for a reference panel. It is also very flexible and allows for any downstream statistical analysis by including the derived SVs as covariates. It has been successfully applied to recover DMPs associated with puberty [25]. SmartSVA can also capture other unmeasured batch effects and possibly population stratification [19, 20]. Though we demonstrated the superior performance of SmartSVA in the context of EWAS, its application can be extended to any other array- or sequencing- based omics data sets. We note that, even if the cell mixture/batch effects is a not a confounding factor, we still recommend running SmartSVA and adjusting for cell mixture/batch effects using the SVs. This practice will improve statistical power by reducing the unexplained methylation variability (error term in the regression model) [26].

SmartSVA, which is built upon classic SVA, assigns weights to the CpG probes when constructing the SVs. These non-informative CpG probes, which are not affected by batch effects, usually receive lower weights. Thus by using the differential weighting scheme, it achieves a similar effect as ReFACTor, which performs PCA on a subset of informative CpG probes. However, compared to ReFACTor, SmartSVA does not require the specification of the number of informative probes used. As we demonstrated in the real data, the number of informative probes does affect the performance of ReFACTor and user diagnostics is thus required to achieve optimal performance for ReFACTor.

Due to the moderate effect size of environmental or biological factors on DNA methylation [4], large-scale EWAS with thousands of or more samples, such as Normative Aging Study [27], have become increasingly popular. A computationally efficient method for cell mixture adjustment is thus needed for large-scale studies. SmartSVA is an order-of-magnitude faster than the classic SVA due to algorithmic improvement and cell mixture adjustment for a large-scale EWAS can be completed within hours.

## Conclusions

With the robustness and power, computational efficiency and minimal required user diagnostics, we believe that SmartSVA will contribute to revealing more reproducible signals from epigenome-wide association studies.

## Methods

### Motivation

Let *A*_*p* × *q*_ = (***a***_1_, ***a***_2_, ⋯, ***a***_*q*_) be the matrix of mean methylation values of *p* CpGs for *q* purified cell types and let *B*_*n* × *q*_ = (***b***_1_, ***b***_2_, ⋯, ***b***_*q*_) be the matrix of unobserved cell compositions of the *q* cell types for *n* samples, where ***a***_*j* = 1,⋯,*q*_ is a column vector of the mean methylation values of cell type *j* for the *p* CpGs and ***b***_*j* = 1,⋯,*q*_ is a column vector of the proportions of cell type *j* for the *n* samples. The observed methylation matrix *Y*_*p* × *n*_ can be expressed as

$$ {Y}_{p\times n}={A}_{p\times q}{B}_{n\times q}^T+{E}_{p\times n}, $$

where *E*_*p* × *n*_ is the error matrix. This motivates us to capture the cell composition through *B* using matrix decomposition methods. When the cell composition varies considerably from individual to individual as observed in real leukocyte counts, the composition variability is expected to account for most of the methylation variability and therefore can be explained by top principal components of the methylation data.

### The SmartSVA algorithm

Surrogate variable analysis (SVA) is an extension of principal component analysis (PCA). PCA seeks to project the data onto a few orthogonal directions so that the variance of the projected data is maximized. The solution of PCA on a data matrix can be obtain using singular value decomposition (SVD)

$$ {Y}_{p\times n}={U}_{p\times n}{D}_{n\times n}{V}_{n\times n}^T, $$

where *U*, *V* are orthonormal matrices and *D* is a diagonal matrix. For methylation data, each column of U could be considered as a methylation ‘eigenarray’, that is, some basic methylation profile shared across arrays. The columns of *V*, called principal components (PCs), contain the loadings for the respective eigenarrays, and may capture the corresponding cell proportions if the eigenarray represents a certain cell type-specific methylation profile. In the presence of other systematic effects on the methylation data, e.g. batch effects and population stratification, PCs may also capture these effects. PC can be written as a linear combination of the methylation vectors for the *p* CpGs

$$ {\boldsymbol{v}}_k={\displaystyle {\sum}_{j=1}^p\frac{u_{j k}}{d_k}{\boldsymbol{y}}_j,} $$

where ***v***_*k*_ is *k*th column of *V*, *u*_*jk*_ is the (*j, k*)th element of *U*, ***y***_*j*_ is a vector of methylation values for CpG *j* and *d*_*k*_ is the *k*th diagonal element of *D*. However, it may be of an advantage to select or weight CpGs to construct the PCs since multiple sources of variation such as cell mixtures may only affect distinct, possibly overlapping, subsets of CpGs. This motivates the development of SVA, which was originally proposed to correct batch effects of unknown sources for gene expression data [19, 20]. The resulted components are called surrogate variables (SVs) in their method, emphasizing the notion that these SVs are surrogates for unmodeled factors. The latest version of SVA requires an iterative algorithm that assigns each probe a weight, which is determined by the probability of the corresponding probe being affected by the unmodeled factors, but not the primary variable of interest. This strategy ensures that the constructed SVs will capture mainly the variation of the unmodeled factors but not the primary variable and is key to retaining power in presence of many signals. Specifically, in each iteration, SVA estimates *p*_*γ*,*j*_ (the probability that the *j* ^*th*^ probe is affected by unmodeled factors) and *p*_*b*,*j*_ (the probability that the *j* ^*th*^ probe is affected by the primary variable conditioned on the unmodeled factors) using an empirical Bayes method based on the current estimate of SVs. The weights are calculated as

$$ {w}_j={p}_{\gamma, j}\left(1-{p}_{b, j}\right),\  j=1,\dots,\ p. $$

Denote *W* = *diag*(*w*_1_, …, *w*_*p*_), SVA then performs a singular value decomposition on the weighted data matrix: *WY* = *UDV* ^*T*^. The algorithm iterates between the two steps to refine the SV estimate for a specified number of iterations. Though the original SVA as implemented in the R Bioconductor package “sva” performs well for most applications, it fails to correct for cell mixtures efficiently under serious cell mixture confounding as demonstrated by simulation. We found that this undesired property was mainly due to a lack of convergence of the solution. We thus propose SmartSVA, an optimized and fast version of SVA, to improve the performance of traditional SVA. SmartSVA has the same input and output as the classic SVA, which takes the methylation data, the number of components and primary variables (in the form of a model matrix) as inputs and outputs the SVs for downstream analysis. SmartSVA involves the following additional steps:

1. Impose an explicit convergence criterion to ensure the convergence of the algorithm instead of using a user-specified number of iterations as in the traditional SVA;
2. Soften the initial estimate of *p*_*b*,*j*_, the probability of being affected by the primary variable conditional on the current SVs, by using a power transform *p*_*b*,*j*_^*α*^;
3. Perform QR decomposition of the model matrix to reduce the computational cost of the most computationally intensive step (calculating F-stat) from *O*(*n* ^2^ *p*) to *O*(*np*), assuming the number of surrogate variables is fixed.

The rationale for step (2) is that SVA starts with performing SVD on the residual methylation matrix, where the effects due to the primary variable are removed. In presence of cell mixture confounding, the initial estimate of *p*_*b*,*j*_ captures the effects of both the primary variable and cell mixtures. Thus the initial *p*_*b*,*j*_ estimate is very inaccurate in highly confounded scenarios and evening out *p*_*b*,*j*_ using a power transform could reach convergence more quickly and significantly speed up the computation. Additional file 1: Figure S7a shows that the number of iteration to reach convergence decreases significantly with smaller *α* values based on a real data set. However, if *α* is very small, it could cause potential local maximums. In such case, the solution is very similar to PCA and there is huge power loss when the signal is dense (Additional file 1: Figure S7b). We thus choose *α* = 0.25 to have a good balance between speed improvement and optimality of the solution. With step (2) and (3), we could speed up the algorithm by an order-of-magnitude.

To determine the number of significant SVs, we propose to use random matrix theory (RMT) [13] since this strategy is adequate for most applications. RMT estimates the number of components by comparing the observed eigenvalues to those of a random matrix counterpart. The number of observed eigenvalues larger than the analytic maximum of the random matrix gives an approximate estimate of the number. Given the increasing sample size of EWAS, the RMT-based method is more appealing due to its computational efficiency than the permutation-based methods.

### Data simulation

Suppose we have *n* samples with a mixture of *q* leukocyte subtypes. We first generate a reference methylation profile by drawing methylation M-values of *p* CpGs from a mixture of three normal distributions with mean *μ*_*j*_^*R*^, standard deviation *σ*_*j*_^*R*^ and mixing probabilities *π*_*j*_^*R*^(*j* = 1, 2, 3), representing hypo-methylated, hemi-methylated and hyper-methylated CpGs. The reference methylation profile represents the methylation of the hematopoietic stem cell. We then generate the methylation profile of two progenitor cells (myeloid and lymphoid progenitor, Fig. 1b) by allowing each progenitor cell to differ in *π* ^*C*^ of the *p* CpGs from the reference with the methylation differences drawn from *N*(0, *σ*_*C*_^2^). Next, for each progenitor cells, we generate four leukocyte subtypes using the same way. For each subtype in each sample, we add various sources of methylation variation to the randomly selected subsets of CpGs. We first add individual-specific methylation differences to *π* ^*I*^ of the CpG sites by drawing the differences from *N*(0, *σ*_*I*_^2^), reflecting the fact that each individual harbors unique methylation signatures due to genetic, environmental and demographic factors. Thus, for any two individuals, they can differ up to 2*π* ^*I*^ of the CpGs for a subtype. To simulate group-specific DMPs between two sample groups (e.g. exposed and unexposed group) for the power study, we add group differences to *π* ^*G*^ of the CpG sites with the differences drawn from *N*(0, *σ*_*G*_^2^). Each leukocyte could have different sets of DMPs with different effect sizes. The observed overall effect size is the weighted average of the effect sizes of individual subtypes with weights being their relative abundances. Without loss of generality, in the simulation, we let the subtypes share DMPs. The cell proportions are simulated from a Dirichlet distribution with mean proportions *π* ^*P*^ and overdispersion parameter *φ*. To make the cell composition a confounding factor, we vary the mean cell proportions of one group with a log2 fold change *f* drawn from *N*(0, *σ*_*F*_^2^) for each subtype. The cell proportions are renormalized to unity sum. The parameter *σ*_*F*_^2^ controls the level of confounding. We then generate the mixed methylation (beta-value) based on the cell proportions. Finally, we add batch effects and measurement errors. Measurement (and other unmodeled) errors are drawn from *N*(0, *σ*_*E*_^2^) for each CpG. For batch effects, we simulate *n*_*B*_ batches while the batch differences are drawn from *N*(0, *σ*_*B*_^2^). All the methylation variability is added to the methylation M-values. The M-values can be converted into beta-values using inverse-logit transformation. Figure 1a gives an overview of the simulation pipeline. For continuous phenotypes, the phenotypes are generated using a standard normal distribution and the log2 fold changes of cell proportion as well as the signals (on M-value) are generated as a linear function of the phenotype. The parameters values used were estimated from a data set of purified cell types (GSE35069) or matched to resemble real methylation data with moderate noise level (Additional file 1: Note1, Fig. 1). In the simulation, we included 10,000 CpG sites to reduce runtime for some computationally intensive methods. The default parameter values are given in Additional file 1: Table S1. All the simulation results were averaged over 100 simulation runs.

### Performance evaluation

We compared the performance of SmartSVA with other reference-free methods in recovering DMPs. Linear regression was used to test for DMPs with the methylation value as the outcome and the group indicator as the covariate, adjusting for PCs/SVs (PCA, SVA, SmartSVA, RefFreeCellMix and ReFACTor) or simulated cell proportions and batch effects (the “Perfect” method). The “Perfect” method offers a hypothetical upper bound in performance and could be used to benchmark other methods. Linear regression was performed on methylation M-values based on the recommendation by Du et al. [28]. Traditional SVA was performed with the default implementation (“sva” R Bioconductor package). For PCA, SVA and SmartSVA, we estimated the number of components using RMT. For ReFACTor, the default parameter setting for ReFACTor (500 informative sites and 5 components) did not control for false positives well in both simulations and real data. To improve its performance, we used 1,000 informative sites and increased the number of components to that determined by RMT for simulations. Using this strategy, the test statistic inflation was generally controlled. FaST-LMM-EWASher [10] was performed using the default parameters without filtering out consistently methylated or unmethylated CpGs since the filtering step could lead to reduced power [7]. RefFreeEWAS and RefFreeCellMix were performed using the default parameters (“RefFreeEWAS” R package) and RMT was used to estimate the number of components. The association *P* values for RefFreeEWAS were calculated based on 100 bootstrap runs.

The false positive (type I error) control was assessed using genomic inflation factor *λ*, observed false discovery rate (FDR) and family-wise error rate (FWER). Genomic inflation factor was defined as the ratio of the median of the empirical distribution of the test statistic to the expected median, thus quantifying the excess false positive rate. Specifically, we first converted the association P values into Chi-square statistic (*χ* ^2^) of 1 degree of freedom and then calculated the genomic inflation factor as

$$ \lambda =\frac{median\left({\chi}^2\right)}{0.456} $$

The observed FDR and FWER were calculated after FDR control (Benjamini-Hochberg procedure) and Bonferroni correction respectively at the nominal level of 5%. Failure to control the FDR and FWER at the nominal level indicates a poor false positive control. Statistical power was assessed using the true positive rate after FDR control and Bonferroni correction. To assess the ability of PCs/SVs in explaining the variability of cell composition, we calculated the multivariate-version of adjusted *R* ^2^ based on a joint analysis of all cell types using canonical correlation analysis (“CCorA” in “vegan” R package). Adjusted *R* ^2^ was used to avoid over-fitting due to potentially a large number of PCs/SVs. All the analyses were performed in R-3.2.0.

### Runtime comparison

We compared the run time of SmartSVA to traditional SVA by conducting a series of simulation experiments on a real data set [1] (GSE42861, *n* = 689, *p* = 485,577). We computed the wall time for each experiment by running the program on an AMD Opteron CPU with 256GB RAM and 16 MB available cache. To have a more meaningful comparison, the wall time included the time used for estimating the number of components by RMT, calculating the SVs and performing association tests using linear regression. Both methods used the same convergence criterion (Spearman’s correlation coefficient > 0.999 between the weights from two consecutive iterations; the stringent criterion is to ensure convergence) instead of using a fixed number of iterations. Two scenarios were independently studied. In the first scenario, the relation between the number of CpG sites in each sample and the runtime of the algorithm was examined. We randomly selected 100 cases and 100 control individuals. The number of CpG sites for each individual was initially set to 4,000 and doubled for each instance of the experiment until reached to 128,000. The second scenario was devoted to studying the runtime variation with respect to the sample size. We sampled *n* = {25, 50, 100, 200, 400 and 600} individuals, and randomly selected 20,000 sites from each individual to create the measurement matrix. We repeated each experiment 20 times.

### Data sets and quality control

To evaluate the performance of the proposed method, we used three real data sets from the study of the methylation change associated with gastric cancer [24] (GSE30601, *n* = 297), serum IgE concentration [22] (*n* = 357) and human aging [23] (GSE40279, *n* = 656). The first two data sets were generated using Illumina HumanMethylation27 BeadChip and the third data set was generated using Illumina HumanMethylation450 BeadChip. Gastric tissue was used to profile methylation for the first study, and peripheral whole blood was used for the last two studies. Probes with detection P values >0.01 in more than 5% of the samples, probes with single-nucleotide polymorphisms (MAF > 0.05, European population, 1000 Genomes Project), and probes on the sex chromosomes were excluded from analysis. We performed the methylation association tests on the raw data since a previous study found that the raw data were already highly reproducible and some normalization approaches might introduce more variability into the data [29]. We did not remove consistently methylated and unmethylated probes since there was no substantial evidence to justify that. For the IgE data set, whole blood cell counts were available for neutrophils, lymphocytes, monocytes, eosinophils and basophils.

### Gene set enrichment

Gene set enrichment for the gastric cancer data set was carried out using DAVID (https://david.ncifcrf.gov/). KEGG pathways and GO biological process (Level 5) were used for enrichment analysis.

### Code availability

R package “SmartSVA” associated with our method is available via CRAN (https://cran.r-project.org/) with documentation and instructions.

## Additional file

Additional file 1: Further methodological details, notes, and additional results. (PDF 3644 kb)

## Abbreviations

DMP: Differentially methylated position
DNA: Deoxyribonucleic acid
EWAS: Epigenome-wide association study
FDR: False discovery rate
FWER: Family-wise error rate
GO: Gene ontology
KEGG: Kyoto encyclopedia of genes and genomes
MAF: Minor allele frequency
PCA: Principal component analysis
RMT: Random matrix theory
SVA: Surrogate variable analysis
SVD: Singular value decomposition
