# Supplementary material for: Fast and robust adjustment of cell mixtures in epigenome-wide association studies with SmartSVA
Source: BMC Genomics. 2017 May 26;18:413. doi: 10.1186/s12864-017-3808-1 (PMC5446715; doi:10.1186/s12864-017-3808-1)
Supplement: Additional file 1: — Further methodological details, notes, and additional results. (PDF 3644 kb) [file 12864_2017_3808_MOESM1_ESM.pdf]

Fast and robust adjustment of cell mixtures in epigenome-wide association studies with SmartSVA

Jun Chen, Ehsan Behnam, et al.

Supplementary Information

Supplementary Note 1
Supplementary Note 2
Supplementary Figure 1
Supplementary Figure 2
Supplementary Figure 3
Supplementary Figure 4
Supplementary Figure 5
Supplementary Figure 6
Supplementary Figure 7
Supplementary Table 1
Supplementary Table 2

Supplementary Note 1. Estimating the methylation difference between cell types and between individuals.

To conduct realistic simulations, it is crucial to obtain an estimate of the proportion of cell-type-specific DMPs (L-DMPs for leukocytes) between cell types and also the distribution of the methylation differences. We use the 450K methylation data set from Reinius et al.[1], which consists of samples of purified neutrophils, CD4+ T cells, CD8+ T cells, B cells, NK (Natural killer) cells, monocytes and eosinophils from six healthy male subjects (GSE35069), to estimate these parameters. No obvious batch effects are observed in this data set and the samples are clustered tightly by leukocyte subtypes. Reinius et al.[1] estimated the number of L-DMP based on a linear model and false discovery rate (FDR) control. At 0.01 FDR, the estimated proportion of L-DMPs ranges from $\sim 5\%$ for closely related subtypes to $\sim 40\%$ for distant subtypes. Due to the small sample size, the method may be underpowered to detect small differences, leading to underestimation. On the other hand, batch effects may confound the differences, resulting in overestimation. Moreover, the estimated distribution of methylation differences is truncated at the decision boundary. To address these limitations, we propose to use a Gaussian mixture model to estimate the proportion of L-DMPs as well as the distribution of the methylation differences between any two leukocyte subtypes from the same individual. The idea behind the mixture model is that the observed methylation difference between subtypes is due to either technical variability or technical plus biological variability, both of which can be modeled as normally distributed. We model the methylation difference $d_{i,k}$(M-value) between two subtypes from the same individual using a two-component Gaussian mixture model:

$$d_{i,k} \sim (1 - \pi^C)N(\mu_E, \sigma_E^2) + \pi^C N(\mu_E + \mu_C, \sigma_E^2 + \sigma_C^2),$$

where $d_{i,k}$ is the methylation difference between two subtypes for subject i and CpG k, $1 - \pi^C$ is the proportion of methylation differences due to measurement errors and/or batch effects, π^C is the proportion due to both measurement errors/batch effects plus real methylation differences, σ_E^2 and σ_C^2 are the variances for measurement errors/batch effects and real methylation differences respectively and μ_E and μ_C are the corresponding means. We include parameter μ_E to allow overall methylation difference between two methylation arrays for two subtypes. This can happen when the bisulfite conversion efficiency is different for the two arrays. μ_C is used to represent the mean methylation difference between these L-DMPs. π^C, σ_C^2 are used to characterize the magnitude of the methylation difference between subtypes with larger values indicating larger difference. We fit the model using Expectation-Maximization (EM) algorithm for every individual and any two-subtype combination. As a byproduct of this process, we also obtain an estimate of the measurement error variance. We also fit a similar mixture model to estimate the methylation difference between two individuals for the same cell subtype. The estimates from the mixture model are used to inform the choices of $\pi^C, \pi^I, \sigma_C, \sigma_I, \sigma_E$ and σ_B (Supplementary Table 4).

Figure SN1.1 shows an example of the fitted model to the methylation differences between monocytes and B cells from one subject and the mixture model fits the data quite well. The distribution of the technical differences has a much smaller variance compared to the distribution of the biological differences. The estimated proportion of L-DMPs between subtypes ranges from 16% to 34% (median 24%) and the estimated standard deviation of the biological differences ranges from 0.77 to 1.31 (median 1.1) on M-values (**Table SN1.1**). Hierarchical clustering based on the proportions or the standard deviations recapitulates the hematopoiesis process, where the granulocytes/monocytes and lymphocytes form two distinct lineages (**Fig. SN1.2**). We also fit the mixture model to the methylation differences between any two individuals for the same leukocyte subtype to estimate inter-individual methylation difference. The estimated proportion ranges from 10% to 16% (median 12%) and the stan-

dard deviation ranges from 0.89 to 1.09 (median 1.0) for different subtypes (**Table SN1.2**). Therefore, the inter-individual difference is much smaller than inter-subtype difference and shows less variability across leukocyte subtypes. The parameter values used in simulations are similar in magnitude to those estimates.

Reference

1. Reinius, L. E. *et al.* Differential DNA Methylation in Purified Human Blood Cells: Implications for Cell Lineage and Studies on Disease Susceptibility. *PLoS ONE* **7**, e41361 (2012).

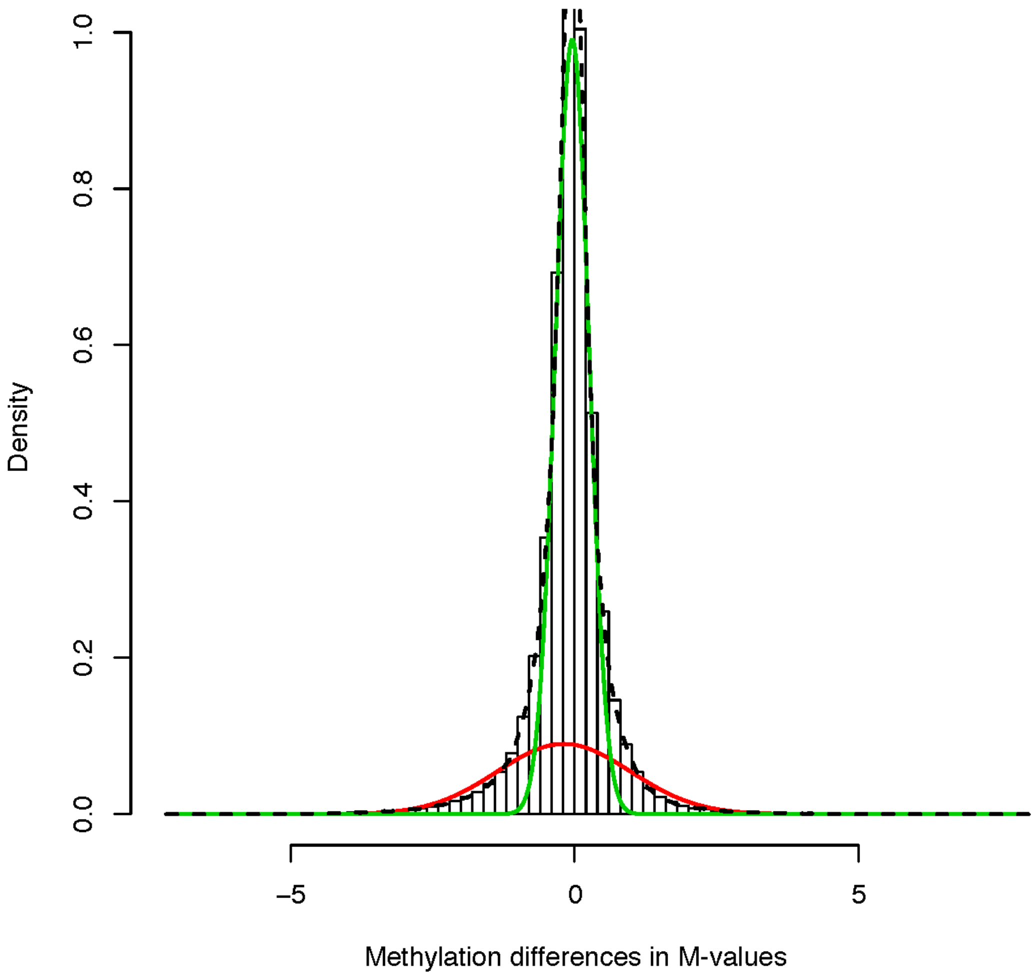

Figure SN1.1 An example of the fitted model based on two-component Gaussian mixture model. The differences in methylation M-value between monocytes and B cells from one subject are used to fit the mixture model. The fitted distribution of the technical differences and the biological differences (plus technical differences) are colored in green and red respectively. The distribution of the technical differences has a much smaller variance compared to the distribution of the biological differences.

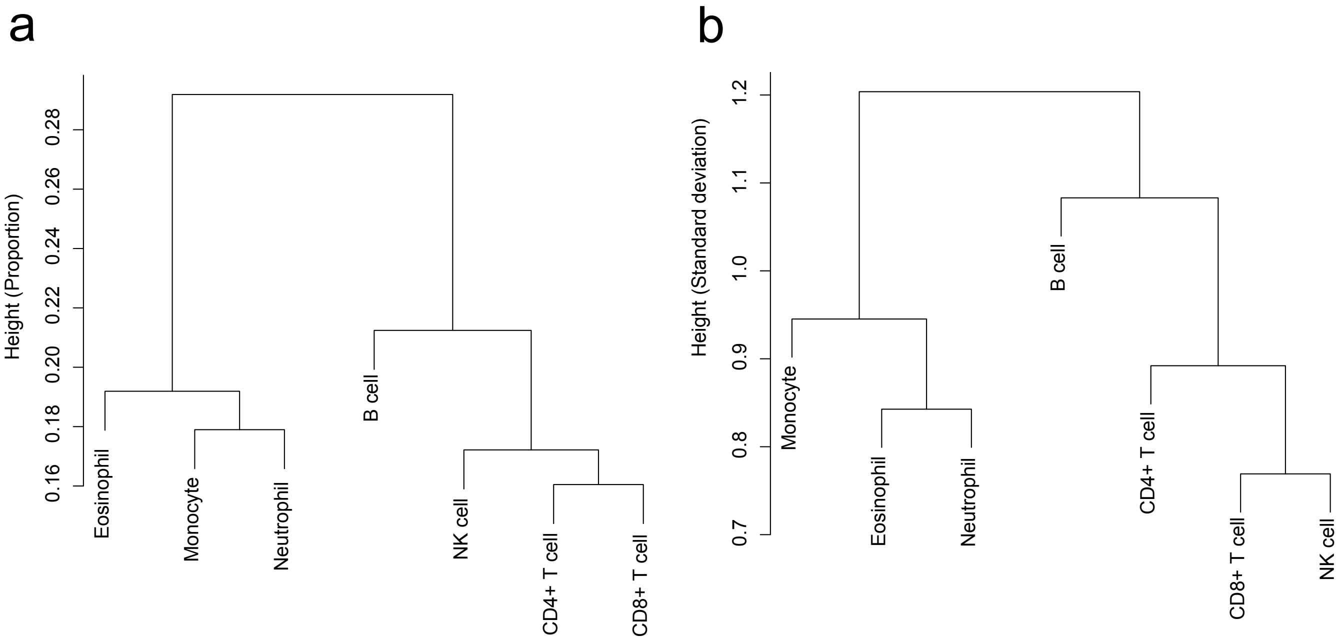

Figure SN1.2 Hierarchical clustering based on the proportions **(a)** and the standard deviations **(b)** of the methylation differences between all leukocyte subtype pairs (**Table SN1.1**). Average linkage is used. Granulocytes/monocytes and lymphocytes form two distinct lineages.

Table SN1.1 The mean proportion of L-DMPs between any two leukocyte subtypes (lower part, blue) and the mean standard deviation of the methylation differences (upper part, red) based on a two-component mixture model. The means are averaged over six biological replicates and standard errors of the mean are given in the parentheses.

	CD4+ T	CD8+ T	NK cell	B cell	Monocyte	Eosinophil	Neutrophil
CD4+ T		0.85(0.03)	0.94(0.04)	1.10(0.03)	1.20(0.04)	1.29(0.04)	1.19(0.05)
CD8+ T	0.16(0.01)		0.77(0.03)	1.10(0.03)	1.19(0.02)	1.31(0.01)	1.21(0.05)
NK cell	0.17(0.01)	0.17(0.01)		1.04(0.03)	1.09(0.02)	1.21(0.03)	1.11(0.03)
B cell	0.22(0.01)	0.20(0.01)	0.21(0.01)		1.15(0.03)	1.29(0.03)	1.22(0.01)
Monocyte	0.23(0.01)	0.34(0.04)	0.34(0.05)	0.28(0.04)		0.98(0.02)	0.91(0.02)
Eosinophil	0.24(0.01)	0.31(0.03)	0.31(0.04)	0.27(0.03)	0.19(0.01)		0.84(0.04)
Neutrophil	0.25(0.01)	0.33(0.04)	0.32(0.04)	0.27(0.03)	0.18(0.01)	0.20(0.01)	

Table SN1.2 The mean proportion of DMPs between individuals (first column) and the mean standard deviation of the methylation differences (second column) for each leukocyte subtype based on a two-component mixture model. The means are averaged over all pairs and standard errors of the mean are given in the parentheses.

	Proportion	Standard deviation
CD4+ T	0.11(0.005)	1.06(0.021)
CD8+ T	0.10(0.006)	1.02(0.014)
NK cell	0.11(0.007)	1.07(0.019)
B cell	0.10(0.005)	1.09(0.028)
Monocyte	0.13(0.002)	0.97(0.005)
Eosinophil	0.15(0.004)	0.99(0.010)
Neutrophil	0.16(0.004)	0.89(0.019)

Supplementary Note 2. The performance of PCA-based methods in dense signal scenarios.

When a large number of CpGs are associated with a primary variable of interest (we denote it as 'exposure' for simplicity) in presence of cell mixture confounding, PCA will fail to work. As the number and/or the strength of real signals increases, PCA starts to capture these signals by some top PCs. Including all the significant PCs as determined by RMT in the regression (PCA1) will over-adjust and most of the real signals will be adjusted away, leading to extremely poor power. Excluding the PC mostly associated with the exposure is also not optimal (PCA2) since in presence of cell mixture confounding it is impossible to distinguish PCs representing the real signals from those representing cell composition. Therefore, PCA on the original methylation data matrix is not a good solution. One may propose to perform PCA on the residual matrix by regressing out the effects due to exposure and include these residual PCs in the regression model (PCA3). This procedure is also problematic since both false and true signals are retained by the regression step and the resulted PCs are orthogonal to the exposure variable and are not able to adjust away the false signals. Hence, PCA on the residuals will retain all the false signals due to confounding. ReFACTor, as a PCA method on a subset of CpG, has the same problem. Though, for a binary exposure variable, selection of informative CpG sites on the controls may alleviate the problem of ReFACTor, the solution cannot be readily extended to a continuous exposure variable. On the other hand, SVA, which constructs the SVs on the original data matrix and up-weights CpGs that are mostly likely to be affected by cell mixtures but not the exposure variable, has the ability to reduce the false positives while retaining the power to a large extent (**Online Methods**). To illustrate the point, we let the exposure affect the methylation of 1,000 CpGs (out of 10,000 CpGs) and increase the abundance of T cell by 50%. We compare the three aforementioned PCA variants (denoted by PCA1, PCA2 and PCA3, respectively) with SmartSVA. **Figure SN2.1a** shows that the averaged ROC curve for PCA1, the PCA on the original data matrix, almost lies on the 45° line, indicating no power to detect signals due to over-adjustment. PCA3, the PCA on the residual data matrix, performs similarly as the unadjusted procedure due to its inability to address confounding. PCA2, the PCA on the original matrix but with the exposure-correlated PC removed performs even worse than unadjustment. The adjusted R^2 values for PCA2 and PCA3 to explain the variance of T cell proportion are much lower than that for SmartSVA, suggesting under-adjustment for these two procedures (**Fig. SN2.1b**). Using Bonferroni correction or FDR control to select the significant DMPs at 5% level, SmartSVA has the overall best performance, identifying more true positives while controlling the number of false positives (**Fig. SN2.1c,d**). In contrast, PCA1 has no power at all and PCA2 and PCA3 produce too many false positives to be of practical use (**Fig. SN2.1d**). PCA2 also shows the largest variability because the removed PC can capture real signals, cell composition or both in each simulation. Given possibly a large number of CpGs loci affected in EWAS, SmartSVA is thus recommended to adjust for cell mixtures.

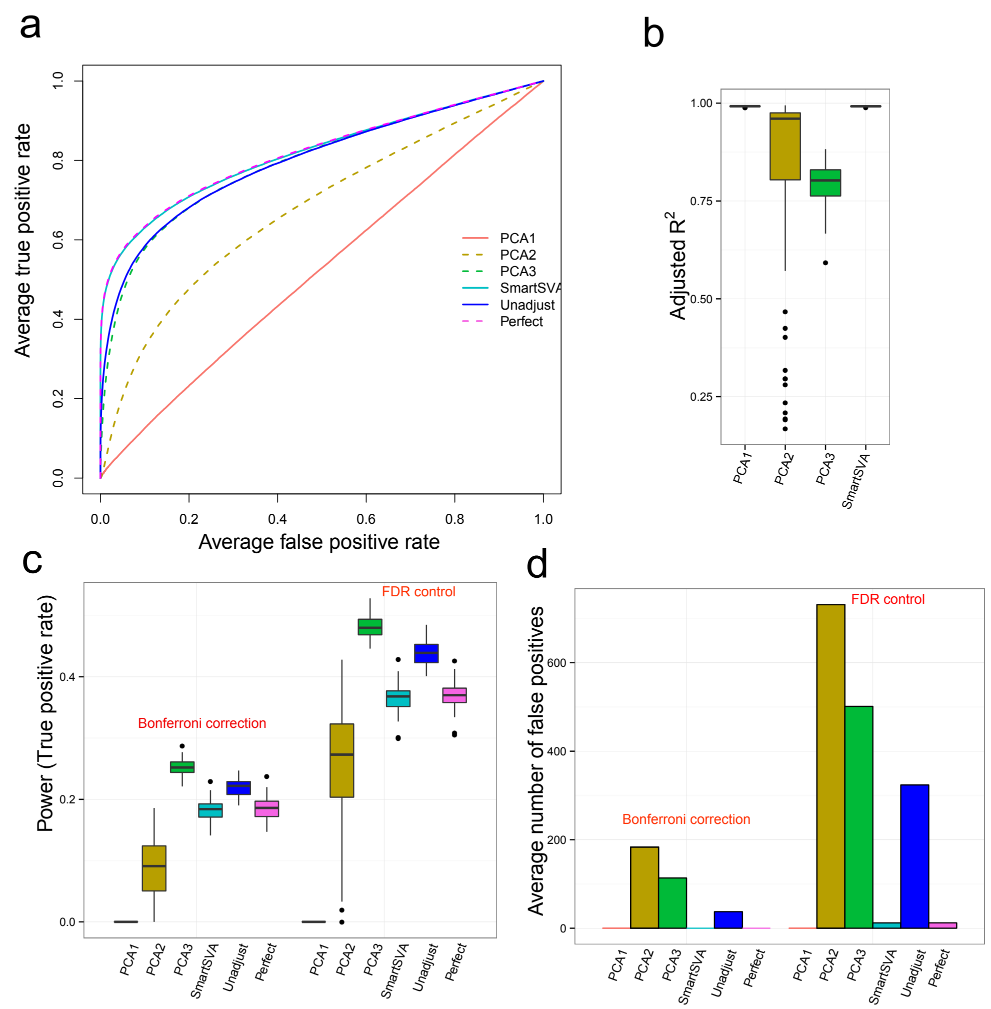

Figure SN2.1 The scenario where PCA breaks down. The exposure affects the methylation of randomly chosen1,000 CpGs and the abundance of T cell. Three PCA methods are compared. PCA1: PCA on the original methylation, PCA2: PCA on the original matrix but with the exposure-correlated PC removed and PCA3: PCA on the residual matrix. All the three PCA methods perform much worse than SmartSVA, demonstrated by smaller AUCs **(a)**. PCA3, the best PCA, is similar to the unadjusted procedure. The adjusted R^2 values for PCA3 and PCA2 to explain the variance of T cell proportion are lower than SmartSVA, indicating under-adjustment for these two procedures **(b)**. When Bonferroni correction and FDR control are used to select the significant DMPs, PCA1 and PCA2 have lower power than SmartSVA **(c)**, while PCA3 identifies too many false signals due to its inability to address cell mixture confounding **(d)**.

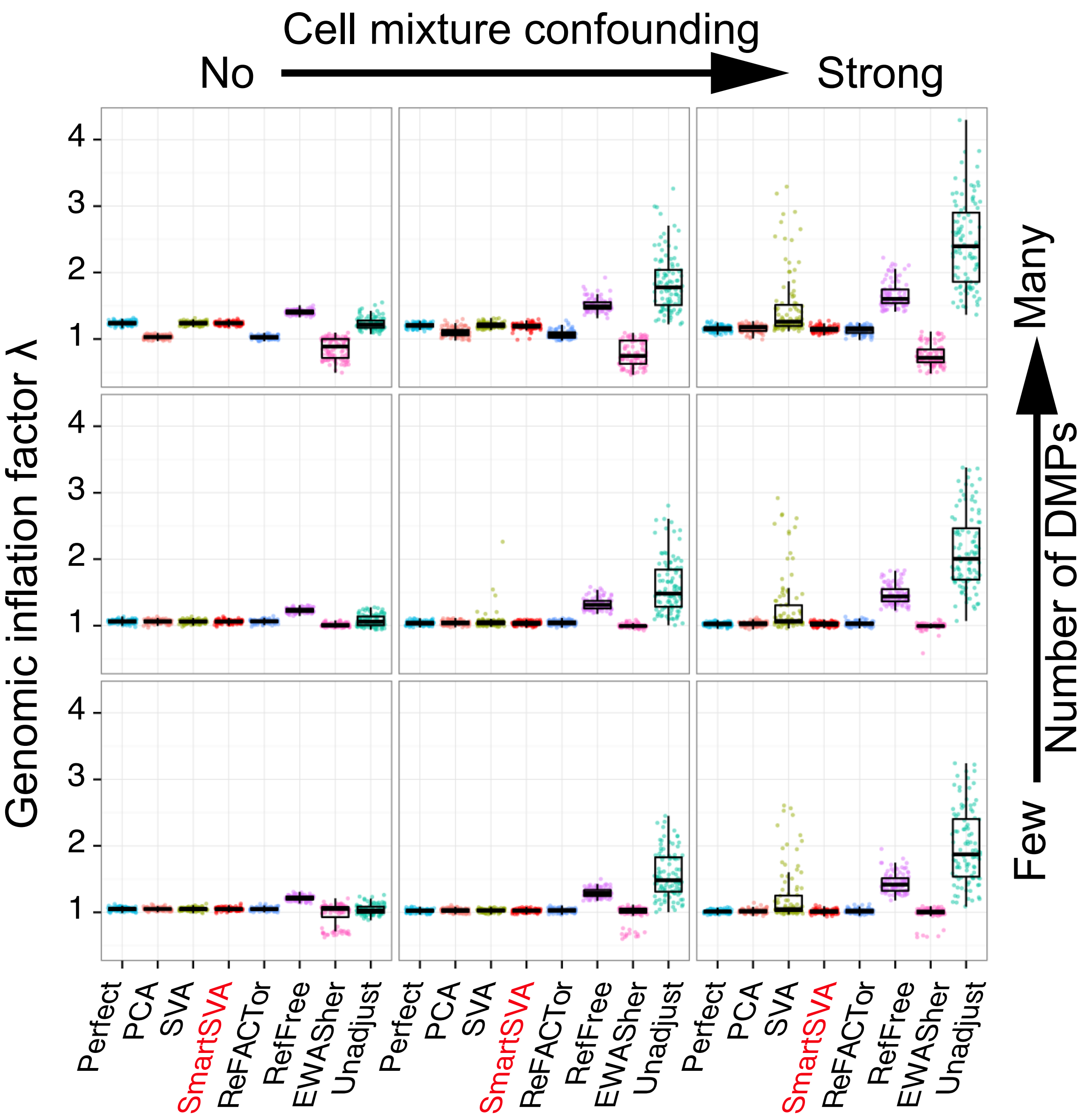

Supplementary Figure 1 Genomic inflation factor λ over all CpGs. Nine scenarios were investigated with different levels of signal density (0.1%, 1% and 10%) and cell mixture confounding (no, moderate and strong). The 'perfect' method, which adjusts for known cell proportions and batch effects, is included to benchmark other methods. As the signal density increases to 10%, an inflated λ over all CpGs has been observed. The inflation is due to a large number of signals since the 'perfect' method, where the adjustment is considered to be sufficient, also shows inflation. Thus forcing λ over all CpGs to 1 will reduce power in dense signal scenarios.

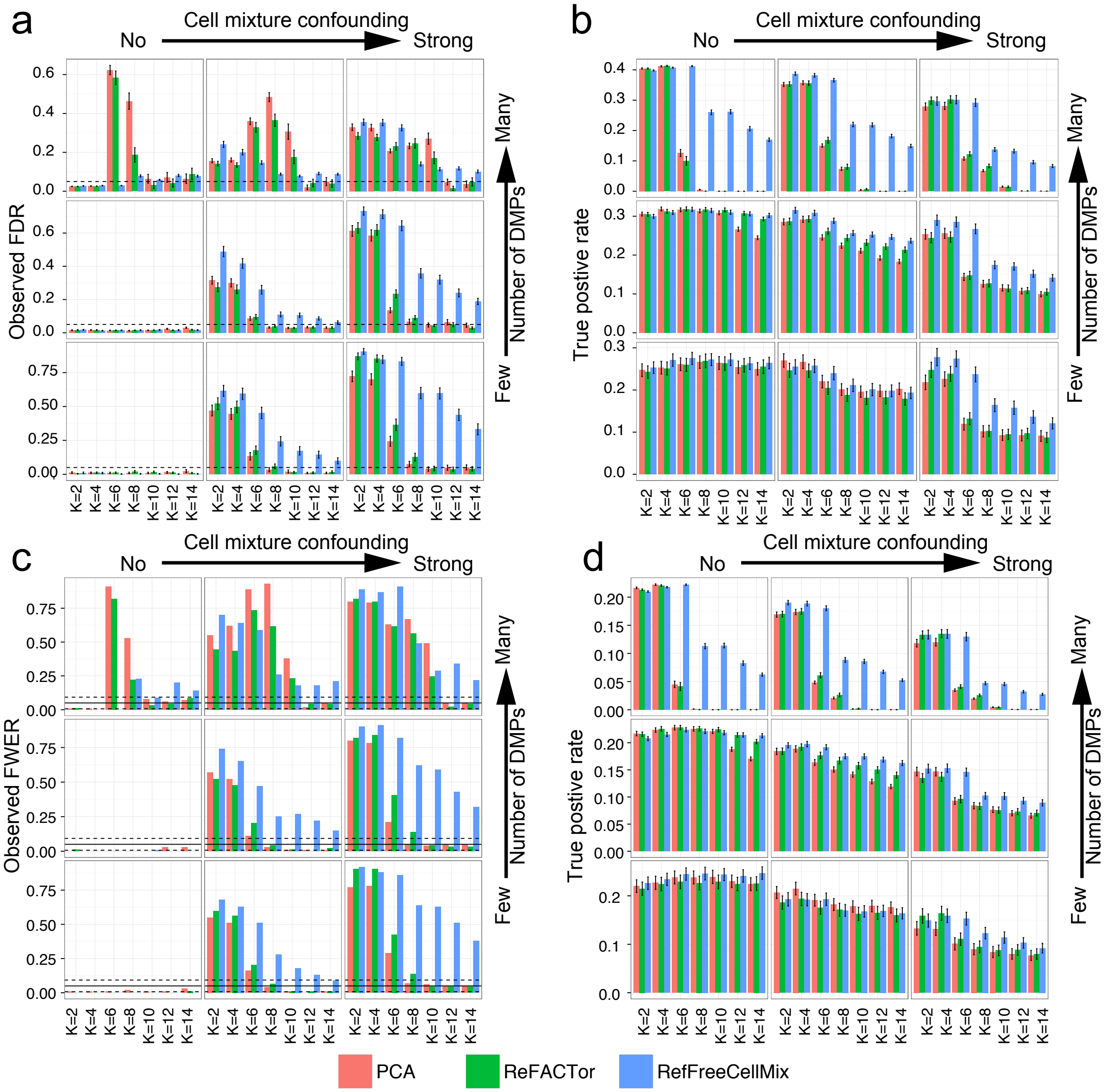

Supplementary Figure 2 The power of ReFACTor decreases with increasing signal densities. Moderate confounding due to cell mixtures was simulated in this example. Performance was evaluated by **(a-b)** the observed false discovery rate (FDR) and true positive rate after FDR control (5% level, dashed line) and **(c-d)** the observed family-wise error rate (FWER) and true positive rate after Bonferroni correction (5% level, solid line; 95% CI, dashed lines). The number of components for ReFACTor was estimated based on RMT. As we increase the signal proportion, the power of ReFACTor decreases significantly, together with reduced ability to control for false positives. In contrast, SmartSVA is very robust, and retains the power irrespective of the signal proportions investigated.

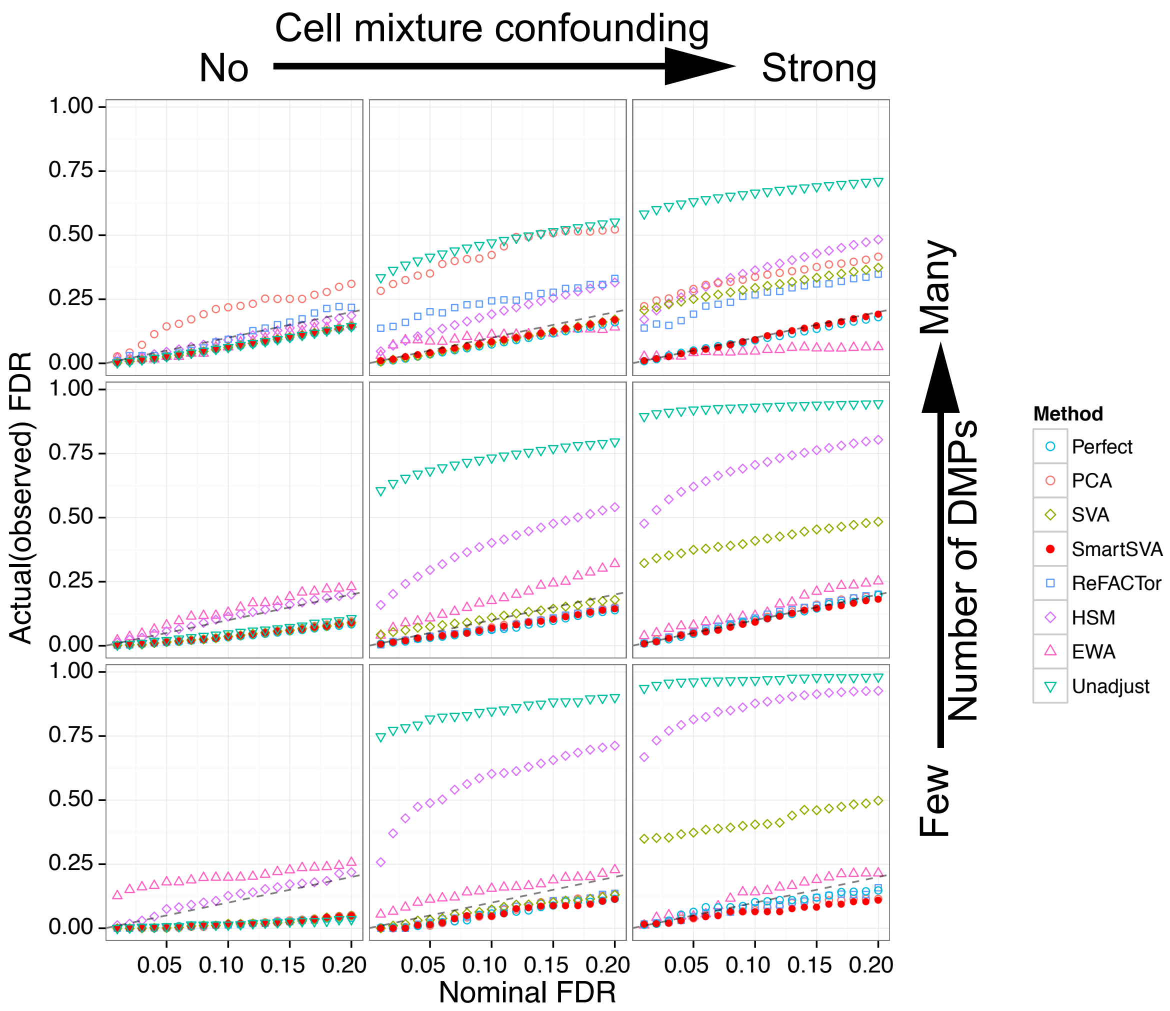

Supplementary Figure 3 SmartSVA controls the FDR under the desired level across different FDR cutoffs. Nine scenarios were investigated with different levels of signal density (0.1%, 1% and 10%) and cell mixture confounding (no, moderate and strong). The 'perfect' method, which adjusts for known cell proportions and batch effects, is included to benchmark other methods. We varied the nominal FDR from 1% and 20%, SmartSVA controls the FDR under the nominal level and has a similar performance as the 'perfect' method across scenarios.

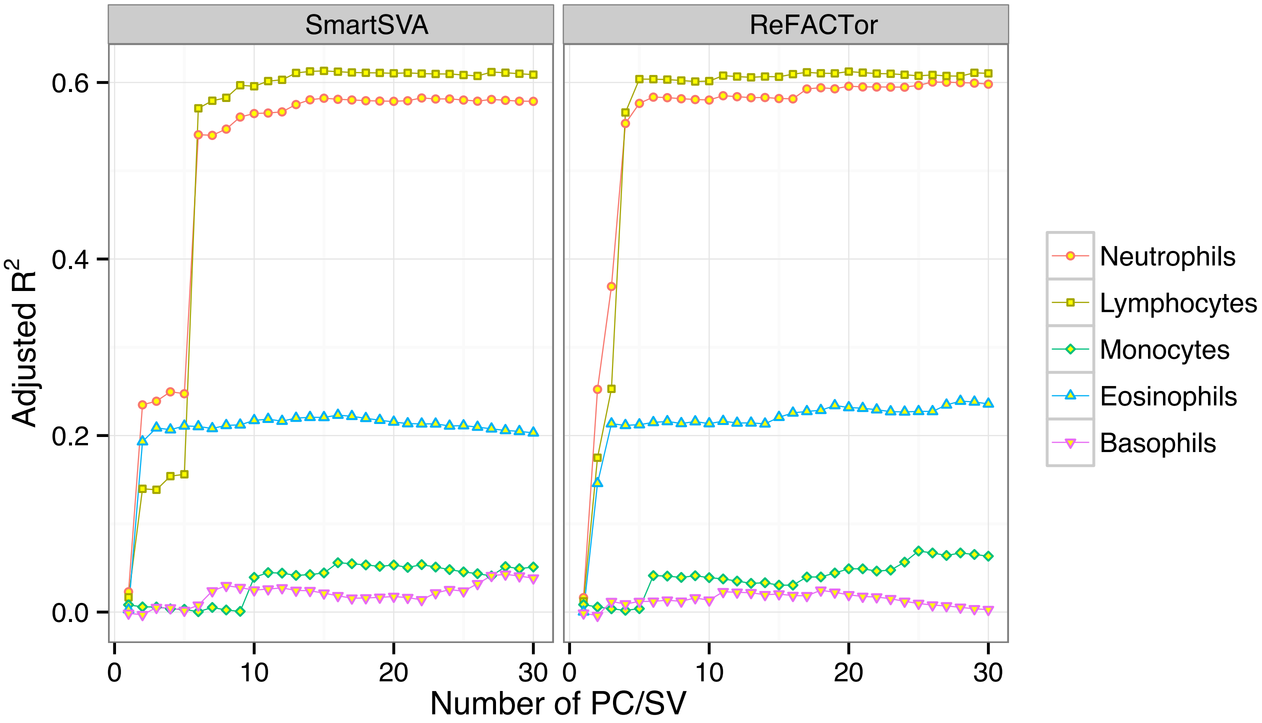

Supplementary Figure 4 The ability of SmartSVA and ReFACTor in capturing cell type composition is similar based on an IgE data set with known blood cell counts ($n = 357$). The fraction of variance explained (adjusted R^2) for each of the cell types with known cell counts is shown.

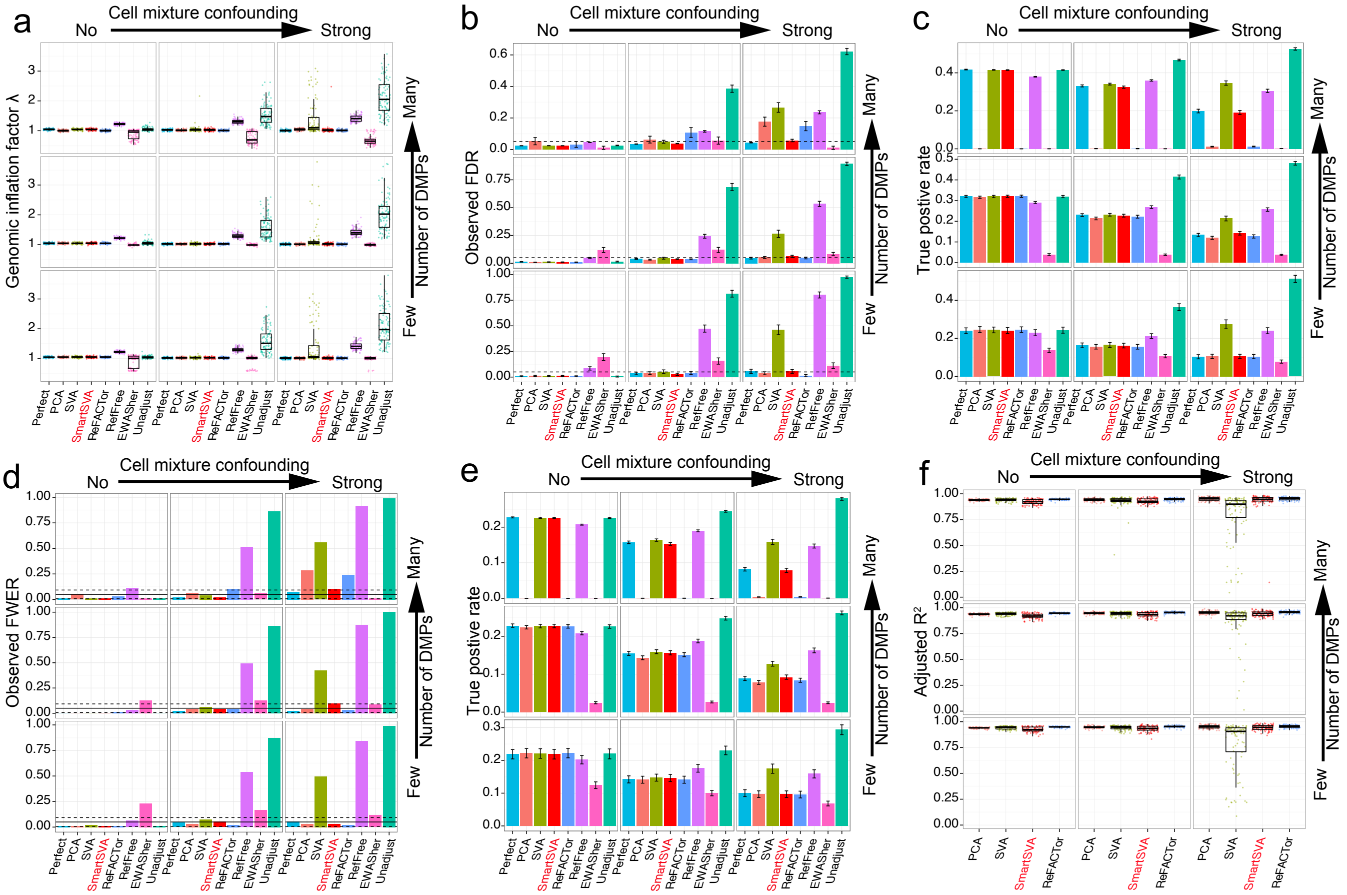

Supplementary Figure 5 Performance comparison of reference-free cell mixture adjustment methods based on simulated data with no batch effects. Nine scenarios were investigated with different levels of signal density (0.1%, 1% and 10%) and cell mixture confounding (no, moderate and strong). The 'perfect' method, which adjusts for known cell proportions and batch effects, is included to benchmark other methods. **(a-f)** Performance was evaluated by **(a)**Genomic inflation factor λ on non-DMPs, **(b-c)** the observed false discovery rate (FDR) and true positive rate after FDR control (5% level, dashed line), **(d-e)** the observed family-wise error rate (FWER) and true positive rate after Bonferroni correction (5% level, solid line; 95% CI, dashed lines) and **(f)** the fraction of cell compositional variability (8 cell types jointly) explained by the components as quantified by adjusted R^2. Error bars represent the standard errors. The results are similar to the those with batch effects. Without batch effects, ReFACTor has slightly better ability to capture cell composition.

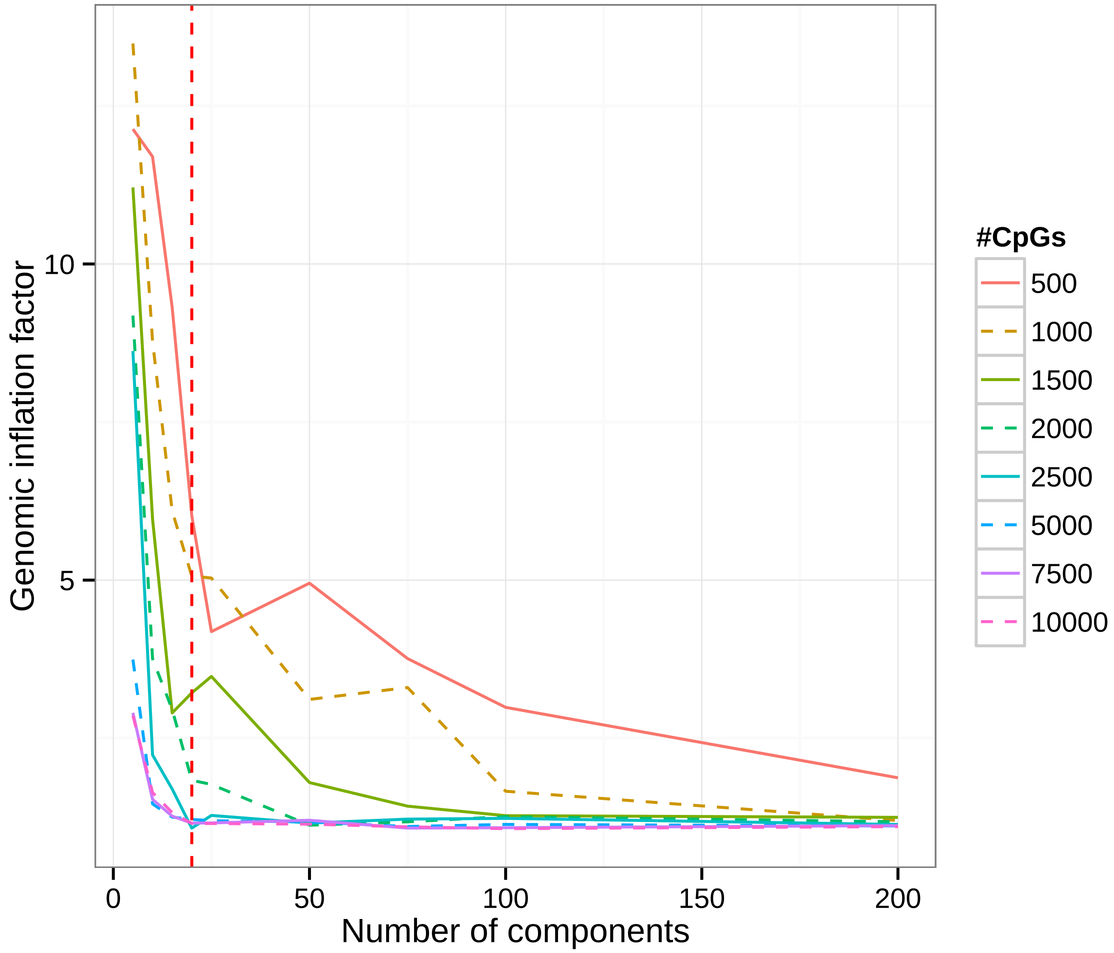

Supplementary Figure 6 The effect of the number of components and CpG sites on the performance of ReFACTor based on the gastric cancer data set. We varied the number of CpGs as well as the number of components, and calculated the genomic inflation factor λ. The default parameter setting ($k = 5, t = 500$) is far from sufficient. We thus used 20 components (indicated by a vertical dashed line) and 2500 CpG sites to control for genomic inflation. The example shows that the manual diagnostics is needed in order to achieve optimal performance for ReFACTor.

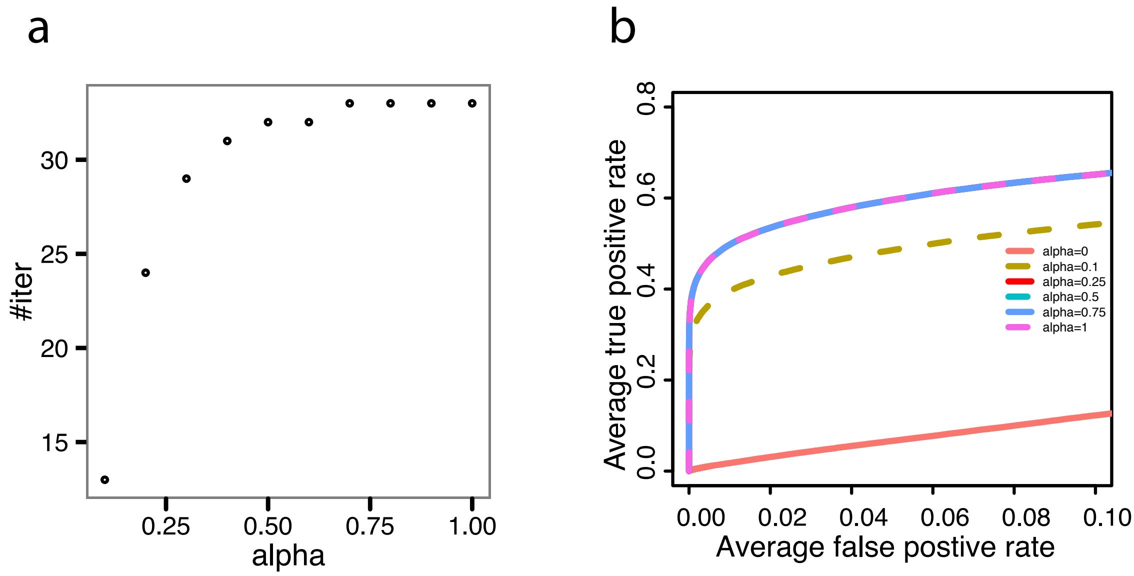

Supplementary Figure 7 The effects of alpha (starting point) on the number of iterations required to reach convergence and performance (ROC analysis). **a**. The gastric data set was used. The number of iterations required increases with alpha. However, when alpha is close to 0, there is a risk of convergence to alocal maximum. **b**. ROC analysis under the simulation setting of dense signal and no confounding (binary phenotype). The false positive rate is plotted against the true positive rate and averaged over 100 iterations. The power is decreased significantly when alpha is small. In such case, the performance is similar to PCA. Thus the exponent alpha=0.25 is a generally safe value while reducing the number of iterations.

Supplementary Table 1 Parameter values used in the simulations.

Notation	Definition	Value
q	Number of cell types	8
n	Sample size (for each group)	100
p	CpG number	10,000
$\mu_1^R, \mu_2^R, \mu_3^R$	Means, standard deviations (SD), mixing probabilities for the three-component normal mixture model for reference methylation profile	-2.5, 0, 2.5
$\sigma_1^R, \sigma_2^R, \sigma_3^R$		1, 1, 1
$\pi_1^R, \pi_2^R, \pi_3^R$		0.4, 0.1, 0.5
π^C	Proportion of cell type-specific DMPs and SD of the methylation differences	10%
σ_C		1
π^I	Proportion of individual-specific DMPs and SD of the methylation differences	6%
σ_I		1
π^G	Proportion of group-specific DMPs (signals) and SD of the methylation differences	0.1%, 1%, 10%
σ_G		0.15
$\boldsymbol{\pi}_L^P$	Mean relative abundances for Lymphoid and Myeloid lineages in controls and precision parameter for the cell-type distribution	(0.09, 0.12, 0.06, 0.03)
$\boldsymbol{\pi}_M^P$		(0.60, 0.06, 0.03, 0.01)
ϕ		25
σ_F	SD of the log2 fold change of cell-type abundance	0, 0.5, 1
σ_E	SD of the measurement error	0.15
n_B	Number of batches and SD of the batch effects	5
σ_B		0.1

Supplementary Table 2. Genomic inflation factor λ's of different methods. The inflation factor λ's (median/IQR) are calculated based on non-DMPs and all DMPs respectively.

Method	Signal (DMPs)[1]	Confounding	λ over non-DMPs[2]	λ over all DMPs[3]
Perfect	Dense	Strong	1.01(0.99-1.02)	1.15(1.12-1.18)
Perfect	Medium	Strong	1.01(0.99-1.02)	1.02(1.01-1.04)
Perfect	Sparse	Strong	1(0.99-1.02)	1(0.99-1.03)
Perfect	Dense	Moderate	1.03(1.01-1.04)	1.21(1.18-1.23)
Perfect	Medium	Moderate	1.03(1.01-1.04)	1.04(1.03-1.06)
Perfect	Sparse	Moderate	1.02(1.01-1.04)	1.02(1.01-1.04)
Perfect	Dense	No	1.04(1.03-1.06)	1.24(1.22-1.26)
Perfect	Medium	No	1.05(1.03-1.07)	1.07(1.05-1.08)
Perfect	Sparse	No	1.05(1.04-1.07)	1.05(1.04-1.07)
Unadjust	Dense	Strong	2.07(1.69-2.42)	2.42(1.96-2.85)
Unadjust	Medium	Strong	2.07(1.62-2.41)	2.1(1.65-2.44)
Unadjust	Sparse	Strong	1.97(1.62-2.49)	1.98(1.62-2.5)
Unadjust	Dense	Moderate	1.41(1.24-1.71)	1.65(1.46-2.01)
Unadjust	Medium	Moderate	1.4(1.24-1.8)	1.42(1.26-1.83)
Unadjust	Sparse	Moderate	1.45(1.26-1.66)	1.45(1.26-1.66)
Unadjust	Dense	No	1.04(0.99-1.08)	1.23(1.18-1.28)
Unadjust	Medium	No	1.03(0.99-1.07)	1.05(1.01-1.09)
Unadjust	Sparse	No	1.03(0.99-1.09)	1.04(0.99-1.09)
PCA	Dense	Strong	1.07(1.04-1.11)	1.16(1.12-1.2)
PCA	Medium	Strong	1.01(1-1.03)	1.02(1.01-1.05)
PCA	Sparse	Strong	1.01(1-1.03)	1.01(1-1.03)
PCA	Dense	Moderate	1.07(1.03-1.1)	1.09(1.04-1.15)
PCA	Medium	Moderate	1.03(1.02-1.05)	1.05(1.03-1.07)
PCA	Sparse	Moderate	1.03(1.01-1.04)	1.03(1.01-1.05)
PCA	Dense	No	1.02(1-1.04)	1.03(1.01-1.05)
PCA	Medium	No	1.05(1.03-1.07)	1.07(1.05-1.08)
PCA	Sparse	No	1.05(1.03-1.07)	1.05(1.03-1.07)
SVA	Dense	Strong	1.08(1.03-1.36)	1.25(1.2-1.58)
SVA	Medium	Strong	1.05(1.02-1.16)	1.07(1.03-1.18)
SVA	Sparse	Strong	1.05(1.01-1.36)	1.05(1.01-1.37)
SVA	Dense	Moderate	1.03(1.01-1.05)	1.21(1.19-1.23)
SVA	Medium	Moderate	1.03(1.01-1.05)	1.05(1.03-1.07)
SVA	Sparse	Moderate	1.03(1.01-1.05)	1.03(1.01-1.05)
SVA	Dense	No	1.04(1.02-1.06)	1.24(1.21-1.26)
SVA	Medium	No	1.04(1.03-1.06)	1.06(1.05-1.08)
SVA	Sparse	No	1.05(1.04-1.07)	1.05(1.04-1.07)
SmartSVA	Dense	Strong	1.01(0.99-1.03)	1.15(1.12-1.17)
SmartSVA	Medium	Strong	1.01(0.99-1.02)	1.02(1.01-1.04)
SmartSVA	Sparse	Strong	1.01(0.99-1.02)	1.01(0.99-1.03)
SmartSVA	Dense	Moderate	1.03(1.01-1.04)	1.2(1.17-1.22)
SmartSVA	Medium	Moderate	1.03(1.01-1.05)	1.05(1.02-1.06)
SmartSVA	Sparse	Moderate	1.03(1.01-1.04)	1.03(1.01-1.05)
SmartSVA	Dense	No	1.04(1.02-1.06)	1.23(1.21-1.26)
SmartSVA	Medium	No	1.04(1.03-1.06)	1.06(1.05-1.08)
SmartSVA	Sparse	No	1.05(1.04-1.07)	1.05(1.04-1.07)
RefFree	Dense	Strong	1.38(1.32-1.5)	1.58(1.51-1.71)
RefFree	Medium	Strong	1.39(1.31-1.51)	1.4(1.33-1.53)

RefFree	Sparse	Strong	1.41(1.31-1.49)	1.41(1.31-1.49)
RefFree	Dense	Moderate	1.28(1.24-1.34)	1.48(1.44-1.55)
RefFree	Medium	Moderate	1.28(1.25-1.33)	1.3(1.26-1.35)
RefFree	Sparse	Moderate	1.29(1.25-1.35)	1.29(1.25-1.36)
RefFree	Dense	No	1.21(1.19-1.24)	1.41(1.38-1.44)
RefFree	Medium	No	1.21(1.19-1.24)	1.23(1.21-1.26)
RefFree	Sparse	No	1.21(1.19-1.24)	1.22(1.19-1.24)
EWASher	Dense	Strong	0.66(0.57-0.79)	0.71(0.62-0.84)
EWASher	Medium	Strong	0.99(0.98-1)	1(0.99-1)
EWASher	Sparse	Strong	0.99(0.98-1.01)	1(0.98-1.01)
EWASher	Dense	Moderate	0.73(0.58-0.95)	0.77(0.62-0.98)
EWASher	Medium	Moderate	0.99(0.98-1)	1(0.99-1)
EWASher	Sparse	Moderate	1.02(0.98-1.05)	1.02(0.98-1.05)
EWASher	Dense	No	0.92(0.71-1)	0.97(0.75-1.01)
EWASher	Medium	No	0.99(0.98-1.03)	1(0.99-1.03)
EWASher	Sparse	No	1.04(0.71-1.1)	1.04(0.71-1.1)
ReFACTor	Dense	Strong	1.04(1.02-1.08)	1.14(1.09-1.18)
ReFACTor	Medium	Strong	1.02(1-1.03)	1.03(1.01-1.05)
ReFACTor	Sparse	Strong	1.02(0.99-1.04)	1.02(0.99-1.04)
ReFACTor	Dense	Moderate	1.02(1-1.06)	1.06(1.02-1.1)
ReFACTor	Medium	Moderate	1.02(1-1.05)	1.04(1.02-1.07)
ReFACTor	Sparse	Moderate	1.03(1.01-1.04)	1.03(1.01-1.05)
ReFACTor	Dense	No	1(0.99-1.02)	1.03(1.01-1.05)
ReFACTor	Medium	No	1.05(1.03-1.06)	1.07(1.05-1.08)
ReFACTor	Sparse	No	1.04(1.03-1.06)	1.05(1.03-1.06)

[1]Dense, medium and sparse signal correspond to 0.1%, 1% and 10% DMPs respectively.

[2]Ideally, λ over non-DMPs should be around 1 regardless of signal density and confounding level. Due to the fact that the data are not generated exactly according to the homoscedastic linear model used in association testing, slight inflation has been observed. Nevertheless, SmartSVA achieves similar λ's as the method 'Perfect', which adjusts for known cell proportions.

[3]For small numbers of DMPs, λ over all DMPs is expected to be around 1 due to the robustness of median statistics. However, as the signal density increases, an inflated λ over all DMPs is expected. Thus in high-density signal scenarios, bringing down λ over all DMPs will reduce power.
